# Supplementary material for: Temporal inversion of the acid-base equilibrium in newborns: an observational study
Source: PeerJ. 2021 Apr 14;9:e11240. doi: 10.7717/peerj.11240 (PMC8052977; doi:10.7717/peerj.11240)
Supplement: Supplemental Information 2 [file peerj-09-11240-s002.pdf]

研究計画書

近赤外時間分解分光モニターによる児の脳循環代謝の病態を明らかにするための研究

研究者：岩田　欧介・松石　豊次郎・岩田　幸子・木下　正啓

【背景と目的】

新生児医療では、中枢神経系障害を有する児の脳循環・酸素代謝の病態を把握し、酸素投与や循環制御を行う脳を中心とした治療指標を設定する事は重要である。また、早産時での慢性肺疾患や未熟児網膜症などの酸素毒性を回避し、脳酸素代謝を考慮した酸素投与の指標作りは重要である。しかし、現在適切な循環管理、酸素投与量を設定するためにベッドサイドで脳循環・酸素代謝量のモニターを行う事は一般臨床で行われていない。1980 年以降生体に安全で透過性が良好な近赤外光を利用した near infrared spectroscopy(NIRS)が新生児の測定に利用されるようになった。最近では、時間分解分光法(Time resolved spectroscopy;TRS)を利用して従来不可能であった定量的測定が可能となり、症例毎の比較が可能となった。今回我々は経時的に TRS を反復測定し、各々の合併症の有無や神経学的予後との関係について比較検討する。

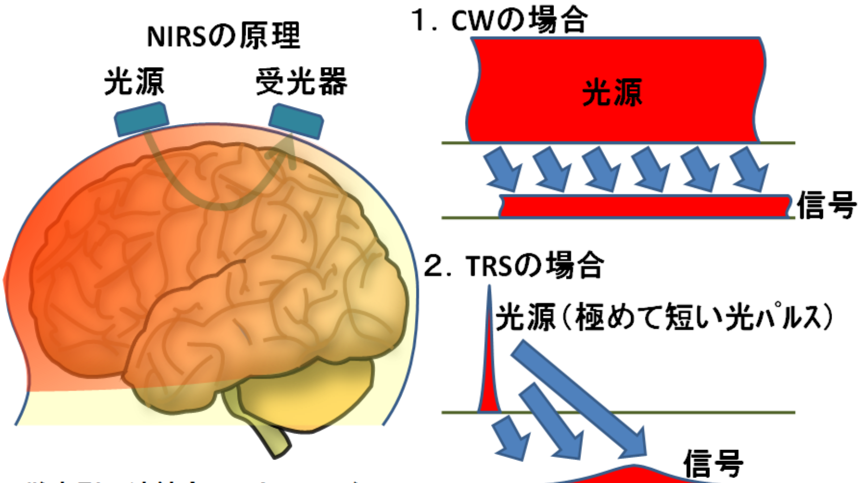

従来型の連続光NIRSとTRSの違い
記録中常に光源から光が出ている従来式NIRSに対し，TRSは非常に短い発光によって，光が通過した組織の厚みに応じて，受光機に到達する時間が異なる．この時間差を解析することで，散乱係数が算出される．

【時間分解近赤外線スペクトロスコピー】

近赤外線スペクトロスコピー（NIRS）は，波長の違う近赤外光の酸化・還元型ヘモグロビン（Hb）による吸光度の違いを利用して，組織中の酸化・還元型 Hb 比を測定する手法で，脳代謝モニターとしての応用が期待されてきた．しかしながら， Hb 酸素飽和度の変化は，血流の変化だけでなく，脳代謝，動静脈血のバランス，動脈血酸素飽和度によっても変化するため，臨床現場では補助的にトレンドを観察する手段として使われるにとどまっている．近赤外光を連続発射する従来式の NIRS に対して，近年応用がすすめられている時間分解法（time resolved spectroscopy, TRS）では，発光時間を極めて短時間に区切ることで，光路長を特定し，酸化・還元型 Hb の絶対量推定が可能となっている．

TRS を用いると，近赤外線の吸収だけでなく，光路長分布が得られるため，組織内の近赤外光散乱係数を算出することができる．光の散乱は，組織の水分含有量や構造の複雑さに強く影響を受けるため，受傷直後の細胞傷害性浮腫や，亜急性期の血管性浮腫，その後の necrosis や apoptosis などによる組織破壊が，散乱係数の変化として観察される可能性が高い．実際に近赤外光の散乱係数は，修正在胎週数の増加に従って増大することが知られている（Ijichi 2005）．われわれのハイリスク新生児におけるパイロット研究からは，散乱係数が ADC と強い相関を見せることが示唆されている．また，われわれの動物モデルによる検討でも，低酸素虚血後の散乱係数の減少が大きい個体において，強い病理組織学的傷害を認めている．

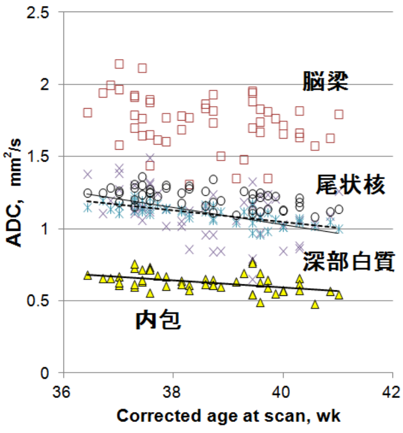

在胎週数と脳内各部位のADC

在胎週数が大きくなるに従って，散乱係数同様に水の拡散係数であるADCも脳構造の複雑化を受けて低下して行く.
Iwata et al. (work in progress)

先行研究とわれわれの施設における基礎データから，十分な症例数における検討で TRS による散乱係数と MRI による ADC が強相関を見せる可能性が高い．これらが証明されれば，散乱係数が超急性期の脳症診断アルゴリズムにおいて，中心的存在となるだけでなく，持続モニタリング可能なツールとして，脳傷害の進行を加味したテイラーメイド治療を実現するツールとなり得ると考えられる．

【研究の方法】
久留米大学新生児センターで入院管理される新生児を対象に，生後 48 時間以内に１回, 以下予定日もしくは退院まで１週間に１回測定を行う．１回の測定は, 前頭部・両側頭部・後頭部で各５０秒程度で行われる．
目標症例数は，成熟新生児　３０名，早産児　３０名，低酸素虚血性脳症児　２０名，先天性心疾患　２０名とする．
TRS 測定値と臨床変量（在胎週数・出生時体重・Apgar score・NICU 入院中の血液ガスおよび血算生化学検査分析結果・レントゲン所見など, 電子カルテから入手可能な情報）の関係にを評価するために，TRS を測定しないコホートにおいても，同様の臨床データの収集を行う．

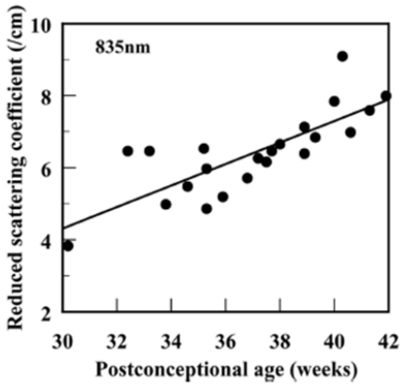

在胎週数と散乱係数
在胎週数が大きくなるにつれて，脳内の構造が複雑化し，近赤外光の散乱が大きくなる傾向が観察されている．
Ijichi et al. 2005 Pediatr Res.

【研究の意義】現在、新生児の中枢神経系の発達や解剖学、生理学的など、超音波検査や MRI、PET などが利用されているが、特に MRI や PET は新生児集中治療室内でのリアルタイムな測定は不可能である。NIRS は非侵襲的でかつベッドサイドでリアルタイムに検査測定が出来るのが特徴である。出生後の児の脳循環や酸素化状態をリアルタイムに把握する事で適切な循環管理や酸素投与量を設定する事が出来るようになると考えられる。

【対象・方法・期間】原則 NICU に入院が必要となった児を対象とする。近赤外時間分解分光モニターにて脳組織酸素飽和度やヘモグロビン濃度など様々な計測を行い、出生後の経時的変化を捉え今後の神経学的予後との関係について明らかにする。研究機関は倫理委員会承認後５年間の予定。臨床変量の比較検討に用いるコホートは，2010 年以降に久留米大学 NICU に入院した新生児約 300 名の電子医療データを後ろ向きに解析する．

【研究参加による利益】神経学的予後との関連が明らかになれば今後の NICU 管理を行う上でこの測定が重要な指標となる可能性がある。

【倫理的側面】TRS 測定を行うスタディコホートにおいては，研究参加者である NICU 入院児の保護者への透明性の高いインフォームドコンセントを行い，書面による同意を得たうえでエントリーする。臨床変量の比較に用いる後ろ向きデータに関しては，診療目的で収集した情報を，個人名を特定できない形で扱うため，個別のインフォームドコンセントは行わず，オプトアウトによる周知を行う．個人情報と連結可能な形で情報処理を行うため，その取り扱いと管理にも万全を期す。本研究では，個人情報を特定可能な形で臨床データとの比較を行うため，検査情報は大学内の決められた場所のみに保管し，個人情報保護のために万全を期す。当該研究に特別な支払いの発生はなく，研究による補償の発生は考えにくい。
研究成果については，研究参加者への個別の確認なしで学術的発表および論文公表を行うことができるよう，説明による同意を得る。研究のために収集した匿名化情報は，研究参加者への個別の確認なしで研究者間で共有し，二次解析を行うことを可能とする。
