# Supplementary material for: Temporal inversion of the acid-base equilibrium in newborns: an observational study
Source: PeerJ. 2021 Apr 14;9:e11240. doi: 10.7717/peerj.11240 (PMC8052977; doi:10.7717/peerj.11240)
Supplement: Supplemental Information 3 [file peerj-09-11240-s003.docx]

**Supplemental Information 1: Background variables of 630 infants who were excluded from analysis.**

| Variables |  |  |
| --- | --- | --- |
| Gestational age (weeks) |  | 36.1 ± 4.4 |
| Body weight at birth (g) |  | 2337 ± 797 |
| Z-score of the above parameters (-) |  | -0.50 ± 1.33 |
| Female sex |  | 289 (46 %) |
| 1-min Apgar score (-) |  | 8 [7-9] |
| 5-min Apgar score (-) |  | 9 [8-9] |
| Caesarean delivery |  | 304 (48 %) |
| Premature rupture of the membranes |  | 121 (19 %) |
| Hypertensive disorders of pregnancy |  | 55 (9 %) |
| Gestational diabetes |  | 150 (24 %) |
| Chorioamnionitis |  | 89 (14 %) |
| Intubation on day 0 |  | 113 (18 %) |
| Non-invasive positive pressure ventilation on day 0 |  | 37 (6 %) |
| Non-invasive positive pressure ventilation on days 5-7* |  | 18 (3 %) |
| Cord blood pH (-) |  | 7.30 ± 0.10 |

Values are shown as mean ± standard deviation, median [interquartile range] or number (%).

*At the time of blood sampling.

See Figure 1 for reasons for exclusion.
