# Supplementary figures and images for: Temporal inversion of the acid-base equilibrium in newborns: an observational study
Source: PeerJ. 2021 Apr 14;9:e11240. doi: 10.7717/peerj.11240 (PMC8052977; doi:10.7717/peerj.11240)

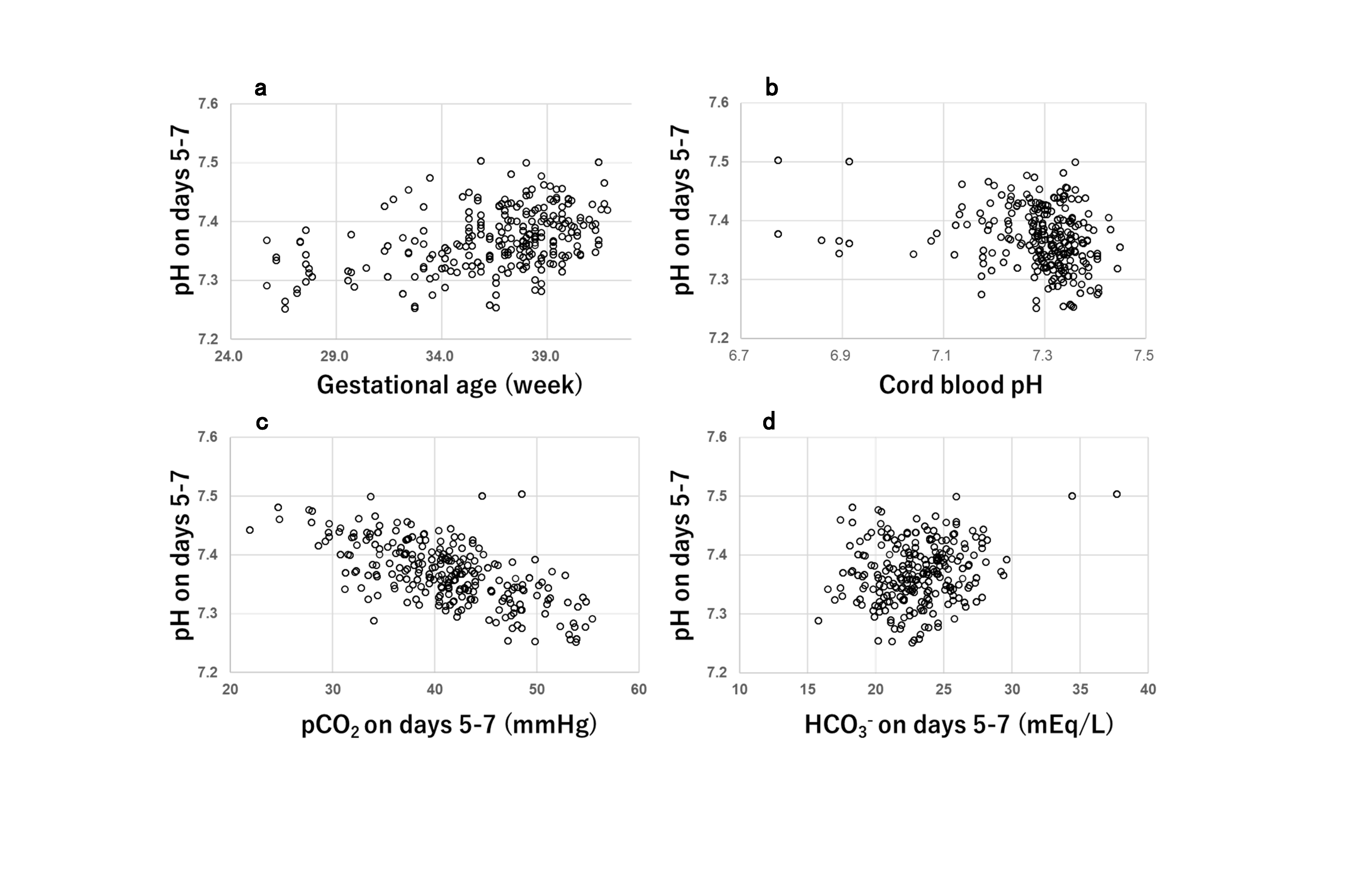

Supplement: Supplemental Information 4 — Representative findings from the univariate analyses, demonstrating the relationships between the higher blood pH on days 5–7 and greater gestational age (a: p < 0.001), lower cord blood pH (b: p < 0.001), lower pCO 2 on days 5–7 (c: p < 0.001) and higher HCO ${}_{3}^{-}$3− on days 5–7 (p = 0.002). [file peerj-09-11240-s004.png]
