# Supplementary material for: Temporal inversion of the acid-base equilibrium in newborns: an observational study
Source: PeerJ. 2021 Apr 14;9:e11240. doi: 10.7717/peerj.11240 (PMC8052977; doi:10.7717/peerj.11240)
Supplement: Supplemental Information 5 [file peerj-09-11240-s005.docx]

**Supplemental Information 3: Dependence of blood pH on days 5-7 on clinical variables: univariate analyses in an alternative, restrictive cohort of infants, who never experienced invasive respiratory support (n = 157).**

|  |  | **Regression coefficient** | | |  |
| --- | --- | --- | --- | --- | --- |
| **Variables** |  | **Mean** | **95% confidence interval** | | ***p*** |
|  |  |  | **Lower** | **Upper** |  |
| Gestational age (weeks) |  | 0.008 | 0.006 | 0.009 | **<0.001** |
| Body weight at birth (per 100g) | | 0.002 | 0.001 | 0.003 | **<0.001** |
| Z-score of above |  | 0.002 | -0.003 | 0.006 | 0.476 |
| Female sex |  | 0.006 | -0.010 | 0.022 | 0.444 |
| Cord blood pH |  | -0.154 | -0.258 | -0.050 | **0.004** |
| 1-min Apgar score |  | 0.005 | -0.002 | 0.013 | 0.176 |
| 5-min Apgar score |  | 0.003 | -0.008 | 0.015 | 0.551 |
| Blood tests on day 0 | Age in hour | 0.000 | -0.001 | 0.001 | 0.535 |
|  | pH | 0.026 | -0.038 | 0.091 | 0.423 |
|  | pCO_2_ (mmHg) | 0.000 | -0.001 | 0.000 | 0.552 |
|  | HCO_3_^-^ (mmol/L) | 0.000 | -0.003 | 0.004 | 0.903 |
|  | Lactate (mmol/L) | 0.003 | -0.001 | 0.007 | 0.194 |
|  | Glucose (mg/dL) | 0.000 | -0.000 | 0.000 | 0.270 |
|  | Na^+^ (mmol/L) | 0.000 | -0.002 | 0.003 | 0.903 |
|  | K^+^ (mmol/L) | -0.004 | -0.013 | 0.005 | 0.365 |
|  | Ca^2+^ (mmol/L) | 0.017 | -0.059 | 0.093 | 0.657 |
|  | Cl^-^ (mmol/L) | -0.002 | -0.003 | 0.000 | 0.058 |
|  | Anion gap (mmol/L) | 0.001 | 0.000 | 0.002 | 0.045 |
|  | Total haemoglobin (g/dL) | 0.003 | -0.001 | 0.007 | 0.164 |
|  | Carboxyl haemoglobin (%) | -0.024 | -0.041 | -0.006 | **0.007** |
|  | Foetal haemoglobin (%) | -0.001 | -0.002 | 0.000 | **0.002** |
|  | Total bilirubin (mg/dL) | 0.002 | -0.001 | 0.005 | 0.181 |
| Blood tests on days 5-7 | Postnatal age (days) | 0.010 | -0.002 | 0.022 | 0.116 |
|  | pCO_2_ (mmHg) | -0.005 | -0.006 | -0.004 | **<0.001** |
|  | HCO_3_^-^ (mmol/L) | 0.001 | -0.003 | 0.004 | 0.664 |
|  | Lactate (mmol/L) | 0.065 | -0.043 | 0.173 | 0.237 |
|  | Glucose (mg/dL) | 0.000 | -0.001 | 0.000 | 0.173 |
|  | Na^+^ (mmol/L) | -0.003 | -0.005 | -0.001 | **0.004** |
|  | K^+^ (mmol/L) | 0.010 | -0.001 | 0.020 | 0.070 |
|  | Ca^2+^ (mmol/L) | -0.079 | -0.159 | 0.002 | 0.056 |
|  | Cl^-^ (mmol/L) | -0.003 | -0.004 | -0.001 | **0.003** |
|  | Anion gap (mmol/L) | 0.001 | -0.003 | 0.004 | 0.695 |
|  | Total haemoglobin (g/dL) | 0.001 | -0.002 | 0.005 | 0.448 |
|  | Carboxyl haemoglobin (%) | 0.003 | -0.015 | 0.022 | 0.718 |
|  | Foetal haemoglobin (%) | 0.000 | -0.001 | 0.001 | 0.938 |
|  | Total bilirubin (mg/dL) | 0.003 | -0.001 | 0.007 | 0.164 |
| Heart rates on day 0 (beats per min) | | -0.001 | -0.002 | -0.001 | **<0.001** |
| Respiratory rates on day 0 (breaths per min) | | -0.001 | -0.002 | 0.000 | 0.320 |
| Heart rates on days 5-7 (beats per min) | | -0.001 | -0.003 | 0.000 | 0.076 |
| Respiratory rates on days 5-7 (breaths per min) | | 0.000 | -0.002 | 0.002 | 0.882 |

Statistical significance was assumed for *p* < 0.01 (indicated in **bold**).
