# Supplementary material for: Temporal inversion of the acid-base equilibrium in newborns: an observational study
Source: PeerJ. 2021 Apr 14;9:e11240. doi: 10.7717/peerj.11240 (PMC8052977; doi:10.7717/peerj.11240)
Supplement: Supplemental Information 6 [file peerj-09-11240-s006.docx]

**Supplemental Information 4: Dependence of blood pH on days 5-7 on clinical variables: univariate analyses in an alternative, expanded study cohort (n = 302).**

|  |  | **Regression coefficient** | | |  |
| --- | --- | --- | --- | --- | --- |
| **Variables** |  | **Mean** | **95% confidence interval** | | ***p*** |
|  |  |  | **Lower** | **Upper** |  |
| Gestational age (weeks) |  | 0.000 | 0.000 | -0.000 | **0.007** |
| Body weight at birth (per 100g) | | 0.003 | 0.002 | 0.004 | **<0.001** |
| Z-score of above |  | 0.001 | -0.004 | 0.006 | 0.703 |
| Female sex |  | 0.006 | -0.010 | 0.021 | 0.486 |
| Cord blood pH |  | -0.115 | -0.165 | -0.065 | **<0.001** |
| 1-min Apgar score |  | 0.005 | 0.001 | 0.008 | 0.015 |
| 5-min Apgar score |  | 0.002 | -0.003 | 0.073 | 0.456 |
| Blood tests on day 0 | Age in hour | 0.010 | -0.046 | -0.009 | 0.003 |
|  | pH | -0.042 | -0.153 | 0.013 | 0.221 |
|  | pCO_2_ (mmHg) | 0.000 | 0.000 | 0.001 | 0.934 |
|  | HCO_3_^-^ (mmol/L) | -0.001 | -0.003 | 0.001 | 0.228 |
|  | Lactate (mmol/L) | 0.042 | 0.025 | 0.058 | **<0.001** |
|  | Glucose (mg/dL) | -0.000 | 0.000 | 0.000 | 0.556 |
|  | Na^+^ (mmol/L) | -0.001 | -0.003 | 0.001 | 0.176 |
|  | K^+^ (mmol/L) | -0.008 | -0.015 | -0.001 | 0.025 |
|  | Ca^2+^ (mmol/L) | 0.063 | 0.023 | 0.102 | **0.002** |
|  | Cl^-^ (mmol/L) | -0.003 | -0.005 | -0.002 | **<0.001** |
|  | Anion gap (mmol/L) | 0.003 | 0.001 | 0.004 | **<0.001** |
|  | Total haemoglobin (g/dL) | 0.003 | 0.001 | 0.005 | 0.001 |
|  | Carboxyl haemoglobin (%) | -0.026 | -0.029 | -0.008 | **0.001** |
|  | Foetal haemoglobin (%) | -0.001 | -0.040 | -0.013 | **<0.001** |
|  | Total bilirubin (mg/dL) | 0.004 | -0.008 | 0.000 | 0.056 |
| Blood tests on days 5-7 | Postnatal age (days) | -0.016 | -0.022 | 0.009 | 0.427 |
|  | pCO_2_ (mmHg) | -0.006 | -0.007 | -0.005 | **<0.001** |
|  | HCO_3_^-^ (mmol/L) | 0.004 | 0.002 | 0.007 | **0.001** |
|  | Lactate (mmol/L) | 0.145 | 0.060 | 0.230 | **0.001** |
|  | Glucose (mg/dL) | 0.000 | -0.001 | 0.000 | 0.047 |
|  | Na^+^ (mmol/L) | 0.003 | -0.005 | -0.002 | **<0.001** |
|  | K^+^ (mmol/L) | 0.002 | -0.007 | 0.011 | 0.711 |
|  | Ca^2+^ (mmol/L) | -0.127 | -0.174 | -0.080 | **<0.001** |
|  | Cl^-^ (mmol/L) | -0.005 | -0.006 | -0.003 | **<0.001** |
|  | Anion gap (mmol/L) | 0.005 | 0.002 | 0.007 | **<0.001** |
|  | Total haemoglobin (g/dL) | 0.005 | 0.002 | 0.008 | **0.002** |
|  | Carboxyl haemoglobin (%) | -0.017 | -0.039 | 0.005 | 0.120 |
|  | Foetal haemoglobin (%) | 0.001 | 0.000 | 0.001 | 0.014 |
|  | Total bilirubin (mg/dL) | 0.005 | 0.003 | 0.007 | **<0.001** |
| Heart rates on day 0 (beats per min) | | 0.000 | 0.000 | 0.001 | **0.001** |
| Respiratory rates on day 0 (breaths per min) | | 0.001 | 0.000 | 0.001 | **0.001** |
| Heart rates on days 5-7 (beats per min) | | 0.000 | 0.000 | 0.001 | **0.004** |
| Respiratory rates on days 5-7 (breaths per min) | | 0.001 | 0.001 | 0.002 | **<0.001** |

The expanded cohort comprises the original study population (n = 200) and those, who remained intubated at the time of blood sampling on days 5-7 (n = 102).

Statistical significance was assumed for *p* < 0.01 (indicated in **bold**).
